# Psychosocial Factors of Health Professionals’ Intention to Use a Decision Aid for Down Syndrome Screening: Cross-Sectional Quantitative Study

**DOI:** 10.2196/jmir.9036

**Published:** 2018-04-25

**Authors:** Samira Abbasgholizadeh Rahimi, Johanie Lépine, Jordie Croteau, Hubert Robitaille, Anik MC Giguere, Brenda J Wilson, François Rousseau, Isabelle Lévesque, France Légaré

**Affiliations:** ^1^ Department of Family Medicine and Emergency Medicine, Faculty of Medicine Université Laval Quebec, QC Canada; ^2^ Canada Research Chair in Shared Decision Making and Knowledge Translation Quebec, QC Canada; ^3^ Université Laval Primary Care Research Centre (CERSSPL-UL) Université Laval Quebec, QC Canada; ^4^ Clinical Epidemiology Program, Ottawa Hospital Research Institute Ottawa, ON Canada; ^5^ Quebec Support for People and Patient-Oriented Research and Trials Units Université Laval Quebec, QC Canada; ^6^ Quebec Centre of Excellence on Aging, Laval University Research Centre on Primary Healthcare and Services Quebec, QC Canada; ^7^ School of Epidemiology and Public Health University of Ottawa Ottawa, ON Canada; ^8^ Department of Molecular Biology, Medical Biochemistry and Pathology, Faculty of Medicine Université Laval Quebec, QC Canada; ^9^ Centre hospitalier universitaire de Québec Quebec, QC Canada

**Keywords:** decision support techniques, Down syndrome, decision making, behavior, intention, physicians, midwifery, surveys, prenatal diagnosis

## Abstract

**Background:**

Decisions about prenatal screening for Down syndrome are difficult for women, as they entail risk, potential loss, and regret. Shared decision making increases women’s knowledge of their choices and better aligns decisions with their values. Patient decision aids foster shared decision making but are rarely used in this context.

**Objective:**

One of the most promising strategies for implementing shared decision making is distribution of decision aids by health professionals. We aimed to identify factors influencing their intention to use a DA during prenatal visit for decisions about Down syndrome screening.

**Methods:**

We conducted a cross-sectional quantitative study. Using a Web panel, we conducted a theory-based survey of health professionals in Quebec province (Canada). Eligibility criteria were as follows: (1) family physicians, midwives, obstetrician-gynecologists, or trainees in these professions; (2) involved in prenatal care; and (3) working in Quebec province. Participants watched a video depicting a health professional using a decision aid during a prenatal consultation with a woman and her partner, and then answered a questionnaire based on an extended version of the theory of planned behavior, including some of the constructs of the theoretical domains framework. The questionnaire assessed 8 psychosocial constructs (attitude, anticipated regret, subjective norm, self-identity, moral norm, descriptive norm, self-efficacy, and perceived control), 7 related sets of behavioral beliefs (advantages, disadvantages, emotions, sources of encouragement or discouragement, incentives, facilitators, and barriers), and sociodemographic data. We performed descriptive, bivariate, and multiple linear regression analyses to identify factors influencing health professionals’ intention to use a decision aid.

**Results:**

Among 330 health professionals who completed the survey, 310 met the inclusion criteria: family physicians, 55.2% (171/310); obstetrician-gynecologists, 33.8% (105/310); and midwives, 11.0% (34/310). Of these, 80.9% were female (251/310). Mean age was 39.6 (SD 11.5) years. Less than half were aware of any decision aids at all. In decreasing order of importance, factors influencing their intention to use a decision aid for Down syndrome prenatal screening were as follows: self-identity (beta=.325, *P*<.001), attitude (beta=.297, *P*<.001), moral norm (beta=.288, *P*<.001), descriptive norm (beta=.166, *P*<.001), and anticipated regret (beta=.099, *P*=.003). Underlying behavioral beliefs significantly related to intention were that the use of a decision aid would promote decision making (beta=.117, 95% CI 0.043-0.190), would reassure health professionals (beta=.100, 95% CI 0.024-0.175), and might require more time than planned for the consultation (beta=−.077, 95% CI −0.124 to −0.031).

**Conclusions:**

We identified psychosocial factors that could influence health professionals’ intention to use a decision aid about Down syndrome screening. Strategies should remind them of the following: (1) using a decision aid for this purpose should be a common practice, (2) it would be expected of someone in their societal role, (3) the experience of using it will be satisfying and reassuring, and (4) it is likely to be compatible with their moral values.

## Introduction

### Background

The decision about whether or not to take a prenatal screening test can be challenging for women [1]. First, the medical information is complex and results are sometimes uncertain [2-4]. Second, to make a good decision, women need to thoroughly understand probabilistic data and the characteristics of the various screening tests such as detection rates [5]. Third, prenatal testing for Down syndrome involves the woman’s personal values, and she may find it difficult to articulate them [1]. Fourth, not knowing the outcome and then finding out the results of the screening may be stressful [6-8] and lead to more difficult decisions. If the results are positive, the pregnant woman will have to decide whether to undergo more invasive diagnostic testing (such as amniocentesis) or not. If these results, too, are positive, she will have to choose between terminating the pregnancy and preparing for a child who will have special needs throughout his or her life [9]. Finally, health professionals’ communication of results is often insufficient, misleading, or negative and they may misjudge women’s values and preferences [5,10-12]. Without sufficient decision support, women in this position may experience decisional conflict, defined as the uncertainty caused by a decision that involves risk, loss, or a challenge to their personal values [13]. Uncertainty may lead to decision regret, which in turn can lead to deteriorating health and perhaps litigation.

In Quebec, Canada, in family medicine groups for example, a first prenatal appointment with a nurse is usually scheduled for a pregnant woman starting from the fifth and sixth week of her pregnancy. The nurse enters information in the patient’s obstetrical file and gives her an information kit that includes the Quebec Ministry of Health and Social Service’s information leaflet on the prenatal test for Down syndrome. Starting from eighth to ninth week, the woman meets the family physician, and the doctor asks her if she wants to take the test.

At this stage, with the help of the physician, the woman should make an informed decision, that is, one that takes into account relevant information about the advantages and disadvantages of all the options and that is in keeping with her values and preferences [14]. However, in reality, studies have shown that in spite of some printed information they are given, few women are making informed decisions about prenatal screening, and health professionals are not engaging pregnant women in shared decision making (SDM) about this decision [12,15].

### Shared Decision Making and Decision Aids

SDM is an interpersonal and interdependent process [16] in which the health professional and patient work together to make informed value-congruent decisions about the patient’s health [17,18]. SDM about this and other health-related decisions not only improves care experiences but also is increasingly recognized as an ethical imperative in health policy and legislation [19]. Patient decision aids (DAs) are decision support tools that help the decision-making process by providing information about treatment or testing options, associated benefits, disadvantages, probabilities and uncertainties, as well as raising the question of values and preferences [20]. Studies have shown that by improving knowledge and allowing patients to make choices that are informed by evidence and by their values, DAs can reduce decisional conflict and indecision [20]. Despite the evidence in favor of SDM and DAs, they are rarely used in practice [21] and even less for prenatal screening decisions.

To increase the use of DAs in making informed decisions about Down syndrome testing, health professionals who are offering women prenatal testing should be targeted as distribution of DAs by health professionals has been shown to be the most promising implementation strategy of SDM to date [22]. According to the theory of planned behavior (TPB) and other sociocognitive theories and models (eg, theoretical domains framework [TDF]), the adoption of a behavior, such as using a DA, is mainly determined by the level of the person’s intention to perform the behavior [23,24]. Some studies have suggested that health professionals’ intention to use a DA would depend on their specialty [25], experience [26], and cultural beliefs [27]. However, these factors are not easily modifiable, whereas according to the TPB, the sociocognitive factors that influence health professionals’ intentions can be modified. Knowledge of these modifiable factors would thus be useful in designing an intervention such as a care plan to foster health professionals’ use of a DA for helping pregnant women make informed decisions about prenatal testing for Down syndrome.

### Objectives

Therefore, we aimed to identify factors influencing health professionals’ intention to use a DA during prenatal visit for decisions about Down syndrome screening.

## Methods

### Study Design and Context

We conducted a Web-based survey of health professionals in the province of Quebec (Canada) using a Web panel and used Checklist for Reporting Results of Internet E-Survey (CHERRIES) to guide reporting of results [28] (see Multimedia Appendix 1). This study was embedded in a larger research initiative called the PEGASUS project (Personalized Genomics for Prenatal Aneuploidy Screening Using Maternal Blood) aiming to validate the performance and utility of noninvasive prenatal testing in the general population. In this larger initiative, our overarching aim was to produce a DA to foster SDM in the context of prenatal screening for Down syndrome. Our study complements a similar survey of the intentions of pregnant women to use a DA for decisions about prenatal screening for Down syndrome [29]. We obtained ethics approval from the research ethics boards of the Centre de Santé et de Services Sociaux de la Vieille-Capitale (#2013-2014-29) and the CHU de Quebec (#B14-02-1929).

### Participants and Recruitment

Prenatal care in the province of Quebec is provided by obstetrician-gynecologists (about 51% of pregnancies), family physicians (about 46%), and midwives (about 3%). These three types of health professionals were eligible to participate, and we expected to recruit a similar proportion of each type as is found in Quebec overall [30]. Recruitment took place from December 18, 2015, to October 4, 2016. Eligible health professionals were as follows: (1) family physicians, midwives, obstetrician-gynecologists, or trainees in these professions; (2) involved in prenatal care; and (3) working in the province of Quebec. We excluded health professionals who were on parental or sick leave and who had participated in a previous phase of the project.

We mandated 2 private firms specialized in polling to program our Web survey and to recruit eligible health professionals in the province of Quebec (Canada). Canada’s health care system consists of 13 (10 provincial and 3 territorial) independent health care systems. In this study, we focused on the province of Quebec, which is the second most populous Canadian province. Once the survey was programmed, we emailed invitations that included an open link to the survey as well as other relevant information. This included: (1) study context, (2) study aim, (3) survey content, (4) ethical approval, (5) funding information, (6) information about researchers, (7) time the survey would take (10 minutes to watch the video and 15 minutes for the questionnaire), (8) honoraria offered to eligible participants (50 Canadian dollars), and (9) coordinator contacts. Invited participants were asked to fill out the questionnaire as soon as possible since the link to the survey would be deactivated when the desired number of respondents was reached. We recruited additional eligible participants by the following means: (1) asking family physicians, midwives, and obstetrician-gynecologists known to the team to forward our invitation (snowball method) and (2) asking 3 relevant provincial health professional associations, which agreed to forward the invitation to their members. By clicking on the survey link, interested participants were directed to the open survey. Once eligibility criteria were confirmed (11 filter questions), eligible health professionals started the voluntary survey. No randomization of items or questionnaires was performed.

### Data Collection

Clear preliminary statements provided information and instructions for the study and enabled participants to confirm their consent. Participating health professionals completed the Web-based survey through 22 Web pages that each included up to 6 items, appearing in the same order for all participants (see Multimedia Appendix 2). Participants were not expected to have experience in the use of a DA, so to have them understand the behavior of interest (Action: *use*; Target: *a DA for prenatal screening for DS*; Context: *during prenatal care visits*), they were invited to watch a 10-minute video that depicted 2 consecutive prenatal care follow-ups during which a pregnant woman, her partner, and a health professional use a DA to decide whether the woman will undergo prenatal screening for Down syndrome. It presented a health professional in a clinic in a scenario that would be relevant to any type of prenatal care provider. The DA is available in Multimedia Appendix 3. The production of the video and the DA followed validated processes used successfully in previous work [31]. After watching the video, eligible health professionals were directed to the survey.

**Figure 1:**
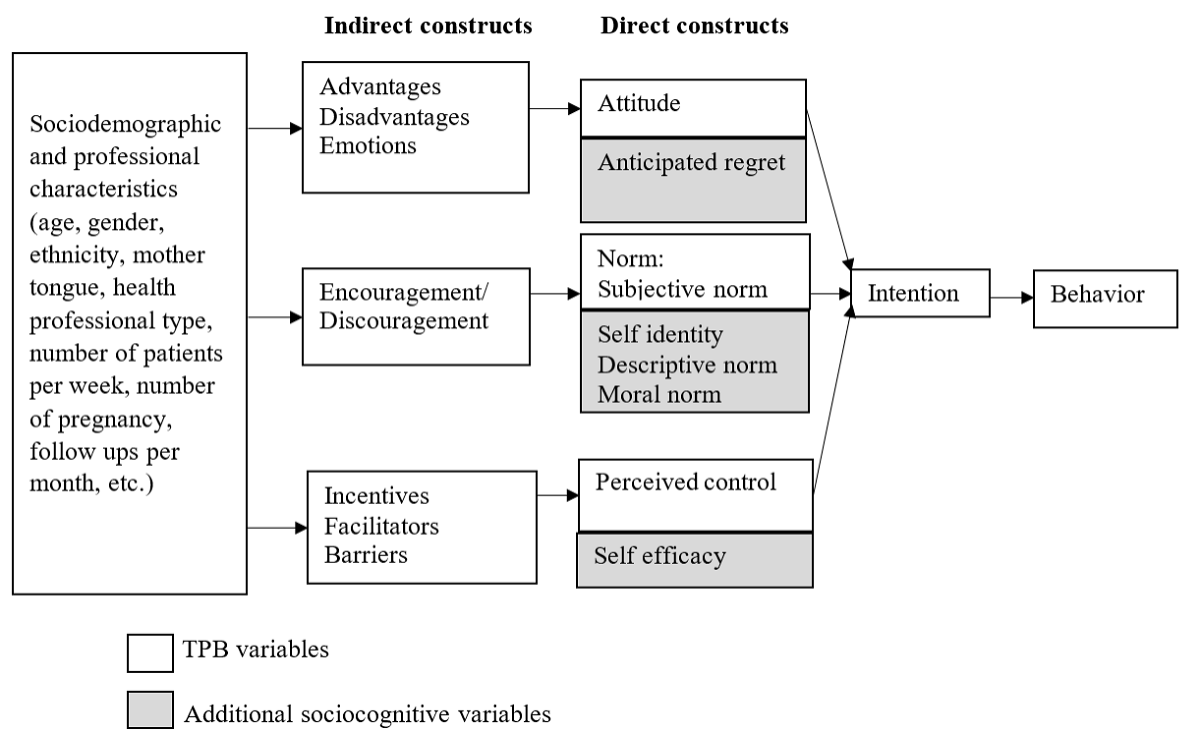
Extended model of behavior change. TPB: theory of planned behavior.

The Web-based questionnaire was developed using constructs that extended the TPB, including some TDF constructs (Figure 1). We used the 3 direct constructs of the TPB: (1) attitude (perceived advantages or disadvantages of adopting the behavior), (2) subjective norm (the perceived social pressure from significant others to perform the behavior), and (3) perceived behavioral control (perceived control over performing the targeted behavior) to assess health professionals’ intention to use a DA for prenatal screening for Down syndrome. We supplemented these with 5 more constructs from theories shown to improve the predictive capacity of the TPB: (1) anticipated regret or potential regret about not having adopted the target behavior; (2) self-identity or one’s image of oneself, reflecting the extent to which a person sees himself or herself as fulfilling the criteria for any societal role; (3) moral norm or one’s perceived moral duties; (4) descriptive norm or the perceived prevalence of the practice; and (5) self-efficacy or perceived ability to enact the behavior [32-34]. We also measured 7 related sets of behavioral beliefs as elicited through semistructured interviews in previous qualitative studies by our team [35], namely, perceived advantages and disadvantages of using the DA, predicted emotions following its use (beliefs underlying *attitude*), encouragement or discouragement (beliefs underlying *subjective norm*), perceived incentives, facilitators and barriers to using the DA (beliefs underlying *perceived control*; Figure 1).

### Questionnaire and Measures

The questionnaire, which was developed both in English and French, included 68 closed items (67 scored on a 7-point Likert-type scale and 1 on a 5-point scale). The questionnaire was pilot tested (2-week test-retest). The aim of the questionnaire was to assess the theory-based factors influencing the use of a DA to decide about prenatal screening [29]. Attitude was measured with 5 items using bipolar adjectives assessing health professionals’ cognitive and affective attitudes (ie, very difficult to very easy, useless to very useful). All other direct constructs were assessed with 3 or 4 items, except anticipated regret with 2 items, and all used opposing outcomes (ie, strongly disagree to strongly agree, very unlikely to very likely). Once data collection was completed, the 2 contracted companies sent us the data anonymously, which were then stored on our secure network (password-protected).

### Sample Size

On the basis of power and sample size determination for linear models [36], we estimated that a sample size of 350 health professionals would be sufficient to detect a partial correlation of .15 between the intention and a model construct, with a power of 80% and a significance level of 5% for each group. To consider the missing data and ensure that our sample was large enough to perform subgroup analyses, we aimed to recruit 380 health professionals (175 gynecologist-obstetricians, 175 family physicians, and 30 midwives, reflecting the proportions of these health professionals who are involved in prenatal care in Quebec province).

### Data Analysis

We used simple descriptive statistics (means, SDs and percentages) to summarize sociodemographics, professional characteristics and sociocognitive variables. For each sociocognitive direct construct and intention, we verified internal consistency by calculating Cronbach alphas. For each sociocognitive construct and continuous sociodemographic variable, we obtained Pearson correlations to assess the strength of their association with intention. For the categorical and dichotomous sociodemographic variables, we performed analysis of variance analyses and *t* tests. We also performed an initial multiple linear regression that included only the TPB variables (attitude, subjective norm and perceived control). Next, we compared the extended TPB model, including the additional variables of anticipated regret, self-identity, moral norm, descriptive norm, and self-efficacy, with the preceding TPB conventional model. We then added the external variables (health professional type and sociodemographics) to the model and used a backward approach in an attempt to obtain an adjusted model with better goodness-of-fit.

To identify significant underlying beliefs, we replaced constructs that significantly determined health professionals’ intention with their associated underlying beliefs (eg, attitude was replaced by its underlying beliefs), and performed the regression model also including all other significant direct constructs. Following a backward approach, we kept significant beliefs (*P*<.05) while keeping the direct constructs in the model.

As our study participants included 3 types of health professionals (family physicians, obstetricians or gynecologists, and midwives), all bivariate analyses involving sociocognitive constructs were conducted on each of the 3 subsamples of health professionals. Due to the small samples and the non-normality of the variables, we used Spearman correlation coefficients to explore the relationship between intention and the sociocognitive constructs per subsample. The multiple regression analysis, including the direct constructs of the extended TPB, was also reproduced in each of these subsamples.

For some of the multiple regression models described earlier, we used deviance and *F* tests to compare nested models and thus identify which one was best. In all regression models, the normality of residuals was satisfying, but their variance was not homogeneous. So we obtained heteroscedasticity-consistent standard errors [37] and reported the corresponding *t* tests for all inferred beta coefficients. All analyses were performed using SAS 9.4 (SAS Institute Inc, Cary, NC).

## Results

### Participant Characteristics

**Figure 2:**
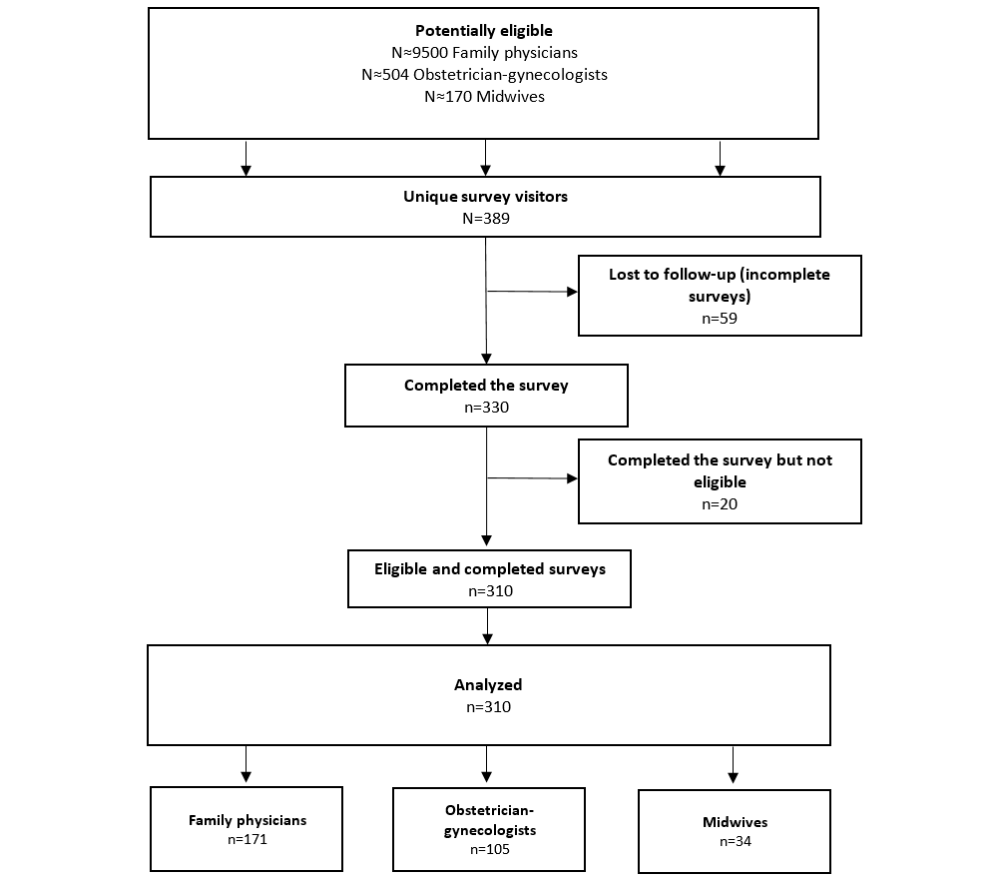
The flowchart of the participants.

Figure 2 illustrates the flow of participants. Following CHERRIES [28], we considered as unique visitors all eligible participants who clicked on the personalized link to take the survey. The completion rate (ratio of users who finished the survey to those who agreed to participate) was 84.8% (330/389). The completion time was not kept for analysis as participating health professionals could stop and restart the survey later, and no data were missing as the Web-based survey did not allow participants to proceed beyond unanswered items. The respondents were not able to review and change their answers. Table 1 shows participant characteristics. Briefly, the mean age of the 310 participating health professionals included in the analyses was 39.6 (SD 11.5) years; 81.0% (251/310) were female; and 92.6% (287/310) were French-speaking. Of the 310 health professionals, 55.2% (171/310) were family physicians, 33.9% (105/310) were obstetricians or gynecologists, and 11% (34/310) were midwives. Surprisingly, although 23.6% (73/310) health professionals reported that they were already aware of a DA for decision making about prenatal screening for Down syndrome, only 40.97% (127/310) of health professionals reported that they knew of any DA for diagnostic or treatment decisions. We found no significant association between health professionals’ knowledge of DAs (in prenatal screening or other contexts) and their intention to use one in the prenatal screening context.

**Table 1:** Health professionals’ characteristics, N=310.

Characteristic		Value
Age in years, mean (SD)		39.6 (11.5)
Number of patients per week, mean (SD)		59.5 (43.1)
Number of pregnancy follow-ups per month, mean (SD)		39.4 (54.1)
**Sex,** **n (%)**		
	Male	59 (19.0)
	Female	251 (81.0)
**Type of health professional, n (%)**		
	Family doctor or family physician or general practitioner	171 (55.2)
	Obstetrician/gynecologist	105 (33.8)
	Midwife	34 (11.0)
**Mother tongue, n (%)**		
	French	287 (92.6)
	English	8 (2.6)
	Other	15 (4.8)
**Ethnicity, n (%)^a^**		
	White or Caucasian	289 (93.2)
	African or African American	2 (0.7)
	Latin American	3 (1.0)
	Arab	7 (2.3)
	South Asian	2 (0.7)
	Southeast Asian	3 (1.0)
	Chinese	2 (0.7)
	Other	1 (0.3)
	I would rather not answer	5 (1.6)
**Prior knowledge about a decision aid for prenatal screening for Down syndrome, n (%)**		
	Yes	73 (23.5)
	No	237 (76.5)
**Prior knowledge about a decision aid regarding another issue, n (%)**		
	Yes	127 (41.0)
	No	183 (59.0)

^a^Percentages add up to 101.5% because participants were allowed to select more than one ethnicity.

### Descriptive and Bivariate Analyses

First, intention and all the 8 direct constructs analyzed showed adequate internal consistency (Cronbach alphas from .75 to .97). Except for anticipated regret (mean score of 3.57 out of 7), mean scores of each construct were higher than 4 out of 7 (4.70 to 5.91) (Table 2), that is, few participants thought they would regret not having used a DA in the context of prenatal screening for Down syndrome.

Bivariate analysis showed that all sociocognitive factors were significantly associated with intention (*P*<.01 to *P*<.001; Table 2). All constructs were also significantly associated with each other (*P*<.05 to *P*<.001) except for descriptive norm and anticipated regret in association with perceived control. In exploring associations between intention and sociocognitive constructs, because the sample was stratified by health professional type we found that, except for perceived control in the midwife sample, all constructs were associated with intention (*P*<.05 to *P*<.001). In order of magnitude, the mean intention scores among health professionals were as follows: midwives 5.78 (SD 0.84), family physicians 5.35 (SD 1.42), and obstetricians or gynecologists 4.91 (SD 1.67).

**Table 2:** Internal consistency of psychosocial constructs and descriptive analyses.

Constructs	Number of items	Cronbach alpha	Mean (SD)
Intention	3	.97	5.25 (1.48)
Attitude	5	.87	5.05 (0.96)
Subjective norm	3	.87	4.74 (1.11)
Perceived control	3	.75	5.27 (1.22)
Self-efficacy	4	.75	5.81 (0.85)
Self-identity	4	.91	5.06 (1.30)
Descriptive norm	3	.91	4.70 (1.44)
Moral norm	4	.90	5.91 (1.01)
Anticipated regret	2	.77	3.57 (1.54)

### Multivariate Analyses

We identified the most significant factors in health professionals’ intention to use the DA. In the first multivariate model, including only TPB variables, attitude (beta*=* 1.104, 95% CI 0.953-1.255) and subjective norm (beta*=*.157, 95% CI 0.028-0.286) were significant factors of health professionals’ intention to use a DA in the context of prenatal Down syndrome screening (Table 3). No sociodemographic variable was added to the model.

In the second multivariate model, based on the extended TPB and thus including the additional variables of self-efficacy, self-identity, descriptive norms, moral norms, and anticipated regret, we found that self-identity (beta=.325, 95% CI 0.186-0.465), attitude (beta=.297, 95% CI 0.168-0.426), moral norm (beta=.288, 95% CI 0.153-0.422), descriptive norm (beta=.166, 95% CI 0.084-0.248), and anticipated regret (beta=.099, 95% CI 0.035-0.164) were significant factors of health professionals’ intention to use a DA in the context of prenatal Down syndrome screening (Table 3). In the TPB-only model, the proportion of explained variance was .64, and in the extended model, it was .80. The increase of 16% in the extended model and the comparison of model deviance shows that health professionals’ intention was better explained when additional sociocognitive variables were included (Δ deviance *F*_5,301_=48.34; *P*<.001); see Table 3).

**Table 3:** Significant factors in health professionals’ intention. TPB: theory of planned behavior; N/A: not applicable.

Construct	TPB, beta (95% CI)	Extended TPB, beta (95% CI)			
	Full sample^a^ (N=310)	Full sample^b^ (N=310)	Obstetricians/ gynecologists^c^ (n=105)	Family physicians/ general practitioners^d^ (n=171)	Midwives^e^ (n=34)
Attitude	*1.104* (0.953-1.255)^f^	*.297* (0.168-0.426)^f^	*.487* (0.237-0.738)^f^	*.197* (0.035-0.359)^g^	*.297* (0.044-0.549)^g^
Subjective norm	*.157* (0.028-0.286)^g^	−.0041 (−0.098 to 0.089)	−0.061 (−0.223 to 0.100)	.018 (−0.118 to 0.154)	−.013 (−0.108 to 0.082)
Perceived control	.044 (−0.054 to 0.142)	.045 (−0.033 to 0.123)	.031 (−0.081 to 0.143)	.041 (−0.079 to 0.161)	−.018 (−0.134 to 0.097)
Self-efficacy	N/A	.193 (−0.01 to 0.395)	−.048 (−0.346 to 0.251)	*.353* (0.078-0.628)^g^	.061 (−0.175 to 0.297)
Self-identity	N/A	*.325* (0.186-0.465)^f^	*.308* (0.055 to 0.56)^g^	*.275* (0.113-0.438)^h^	*.334* (0.146-0.521)^h^
Descriptive norm	N/A	*.166* (0.084-0.248)^f^	*.201* (0.088 to 0.313)^f^	*.148* (0.030-0.266)^g^	.056 (−0.047 to 0.158)
Moral norm	N/A	*.288* (0.153-0.422)^f^	*.393* (0.162 to 0.624)^h^	*.224* (0.051-0.396)^g^	.233 (−0.051 to 0.517)
Anticipated regret	N/A	*.099* (0.035, 0.164)^h^	.072 (−0.028 to 0.172)	*.155* (0.071-0.238)^f^	−.02 (−0.134 to 0.093)

^a^R^2^=.644; deviance=241.80.

^b^R^2^=.803; deviance=134.12; *F*_5,301_=48.34, *P*<.001 for comparison with TPB model deviance.

^c^R^2^=.869; deviance=37.85.

^d^R^2^=.766; deviance=80.37.

^e^R^2^=.758; deviance=5.70.

^f^*P*<.001.

^g^*P*<.05.

^h^*P*<.01.

**Table 4:** Significant beliefs of health professionals. N/A: not applicable.

Construct	Underlying belief	Beta (SE)	*P* value
Attitude	Advantages: Using a decision aid would promote decision making	.117 (.037)	.002
	Emotions: Using a decision aid would reassure me	.100 (.038)	.01
	Disadvantages: Using a decision aid might require more time than planned for the consultation	−.077 (.024)	.001
Self-identity	N/A	.366 (.070)	<.001
Moral norms	N/A	.319 (.064)	<.001
Descriptive norms	N/A	.209 (.043)	<.001
Anticipated regret	N/A	.094 (.031)	.003

Analyses of the extended model within the strata of health professional type suggested some differences in the size effects of the factors, but due to collinearity issues we were not able to assess the statistical significance of these differences. For the obstetrician or gynecologist subgroup (N=105), we found that, in order of importance, attitude (beta=.487, 95% CI 0.237-0.738), moral norm (beta=.393, 95% CI 0.162-0.624), self-identity (beta=.308, 95% CI 0.055-0.560), and descriptive norm (beta=.201, 95% CI 0.088-0.313) were significant factors of intention. For the family physician subgroup, we found that self-efficacy (beta=.353, 95% CI 0.078-0.628), self-identity (beta=.275, 95% CI 0.113-0.438), moral norm (beta=.224, 95% CI 0.051-0.396), attitude (beta=.197, 95% CI 0.035-0.359), anticipated regret (beta=.155, 95% CI 0.071-0.238), and descriptive norm (beta=.148, 95% CI 0.030-0.266), were significant factors of intention. Finally, for the midwife subgroup, we found that self-identity (beta=.334, 95% CI 0.146-0.521) and attitude (beta=.297, 95% CI 0.044-0.549) were significant factors of intention (Table 3).

Attitude was the only significant construct among TPB variables. Thus, to identify significant underlying beliefs, we performed an additional multiple regression model where attitude was replaced with its underlying beliefs (Table 4). From this, we found 3 significant beliefs related specifically to the attitude construct, namely, that the use of a DA (1) would promote decision making (beta=.117, 95% CI 0.043-0.190), (2) would reassure health professionals (beta=.100, 95% CI 0.024-0.175), and (3) might require more time than planned for the consultation (beta=−.077, 95% CI −0.124 to −0.031).

## Discussion

### Principal Findings

With the aim of helping health professionals to support women to make informed, value-congruent decisions about prenatal testing, we identified psychosocial factors influencing the intentions of midwives, family physicians, and obstetricians or gynecologists to use a DA during a prenatal visit for decisions about Down syndrome screening. We found the following: (1) less than half of the health professionals were aware of DAs for contexts other than prenatal screening, and few of them knew of any DA for prenatal screening for Down syndrome; (2) all psychosocial measures except for anticipated regret scored high; (3) overall intention was high among health professionals but varied across the type of health professional, and attitude, self-identity, descriptive norm, moral norm and anticipated regret were all associated with intention to use a DA for prenatal screening among all types of health professionals; and (4) 3 significant beliefs related to attitude in all groups were that the use of a DA would promote decision making, would reassure health professionals, and might require more time than planned for the consultation. These results lead us to make the following four main observations.

First, health professionals do not know enough about DAs in any context, including in the prenatal screening context. Studies show the important role of health professionals in the delivery of DAs [22,38]. Our results, showing that more than half of all health professionals surveyed had never come across any DAs, indicate that more needs to be done to distribute DAs in health care systems and make health professionals aware of them. This concurs with results of qualitative studies on health professionals’ attitudes to DAs, suggesting that their lack of awareness of the existence of DAs was a major barrier to their use [39,40]. In addition, we observed that the video was needed for showing health professionals what a DA was, as well as how it can be used during a consultation, to ensure that they had a clear idea of what they were being surveyed about. This result also suggests that another criterion could be added to the International Patient Decision Aids Standard, that is, that the purpose and potential use of the DA is comprehensible to health professionals. Further studies assessing factors influencing the use of DA should start with asking about target participants’ awareness of DAs and make sure they know what a DA is and how they can be used in clinical settings.

Second, few participants thought they would regret not having used the DA. Regret is “a comparison-based emotion of self-blame, experienced when people realize or imagine that their present situation would have been better had they decided differently in the past” [41]. One of the conditions that determines anticipated regret is when the preferred option is not necessarily superior to other options [41,42]. It could be that health professionals feel they are already engaging their patients adequately in the decision about prenatal testing, and that the option of using the DA is not greatly superior to their own efforts. This is supported by studies showing that health professionals tend to think they are engaging in SDM more than their patients think they are [43]. Further investigation is required to identify the main reason for health care professionals reporting they do not anticipate regret for not using a DA in this context.

Third, intention to use a DA was high overall among health professionals. These results are congruent with previous studies showing high levels of intention among health professionals to engage in SDM in clinical contexts (including prenatal screening) [44]. Interestingly, some of our previous work has shown that pregnant women also have high levels of intention to use a DA for Down syndrome screening decisions [29,45]. Together, these results suggest that both health professionals and pregnant women seem inclined to use a DA in Down syndrome prenatal screening, and that lack of intention among either health professionals or pregnant women is not the cause of failure to implement DAs in this context. Although similar factors influenced their behavior (attitude, moral norm, descriptive norm, and anticipated regret), future interventions will need to be tailored to each member of the dyad.

However, although overall intention was high, we also observed that health professionals’ intention to use a DA for prenatal Down syndrome screening may differ by type: midwives had the highest intention and obstetrician or gynecologists the lowest. Our results are the first to document that this intention varies across types of health professional. The variation could be due to obstetricians or gynecologists feeling that they have less time to enter into a lengthy discussion about Down syndrome prenatal screening. One study highlighted the importance of using DAs in a flexible manner, that is, adapted to timing appropriate to the needs of different types of health professionals [40]. The variation in intention could also be explained by their different views regarding their role and responsibilities [46-48]. For instance, although midwives see their role more as one of “providing information” and letting the patient decide, physicians more often consider their role as that of an “advisor” or an “educator” and feel the decision is their responsibility [48]. These differences reflect differences in training, philosophy, professional culture, and practice among the 3 types of health care professionals [46,47]. Researchers, curriculum developers, and providers of continuing education should adapt SDM training to the different types of health professionals.

Fourth, our findings indicate that health professionals’ intention to use a DA in this context is determined by, in order of importance, their image of themselves as fulfilling a societal role (*self-identity*); the consequent advantages, disadvantages, and emotions they perceive (*attitude*); its compatibility with their moral values (*moral norm*); their perception of how much other health professionals use DAs (*descriptive norm*); and the regret they perceive they might feel if they do not use it (*anticipated regret*). These results align with our earlier qualitative results regarding these factors [35]. Interventions to foster the use of a DA for Down syndrome prenatal screening by health professionals should address these factors, for example, by introducing the advantages of using the DA (attitude), spreading the culture of using DAs through social media (moral norms, self-identity), presenting the use of DAs as a desirable practice (descriptive norm), and suggesting to health professionals that they might regret not using it (anticipated regret).

In addition, three significant underlying beliefs were identified. One was the belief that it might require more time than planned for the consultation. In a 2017 Cochrane systematic review of DAs, 8 out of 10 studies that measured consultation length reported no significant difference for the DA group compared with the control group [20]. Further studies are required to investigate if the use of DA takes more time or not. Key statements regarding these 3 salient beliefs could be added to SDM training materials (eg, continuing professional development course material, videos) to increase health professionals’ intention to use DAs in clinical practice.

### Limitations

This study has a few limitations. First, those who agreed to participate may have been more inclined to use DAs than those who did not. Second, the study targeted health professionals in Quebec, that is, in one health care system, so we cannot infer that our results are applicable to other health care systems including those in other Canadian provinces and territories. Although our results can inspire other efforts, interventions need to be adapted to each prenatal care pathway. Third, the invitation to complete the survey was sent to health professionals’ organizations and personal email lists, and so calculation of a precise view rate (ratio of unique survey visitors to unique receivers of survey invitation) was not possible. Finally, we focused on 3 types of health professionals who are directly involved in the decision-making process with couples (family physicians, obstetrician or gynecologists, and midwives), although other health professionals, such as nurses and geneticists, are also likely to be involved at some stage of prenatal follow up and in prenatal screening decisions. Further studies will be needed to elucidate their specific roles and beliefs.

### Conclusions

On the basis of a theoretical approach to behavior change and following best practices for conducting Web-based surveys [28], this study identifies psychosocial factors that could influence health professionals’ intention to use a DA for helping pregnant women make informed decisions about Down syndrome screening, and suggests which factors will need to be addressed in an intervention to increase their intention. An earlier study investigating factors influencing intention to use such a DA among pregnant women observed high levels of intention, and in this study too, in general, all types of health professional showed high intention. These combined results, as well as our new detailed information on what behavioral factors to address, lead us to suggest that the time is ripe for implementing an intervention to foster DA use in this context.

## Appendix

Multimedia Appendix 1 Checklist for Reporting Results of Internet E-Surveys (CHERRIES).

Multimedia Appendix 2 Online questionnaire.

Multimedia Appendix 3 The decision aid for prenatal screening for Down syndrome.

## Abbreviations

SDM: shared decision making
DA: patient decision aid
TPB: theory of planned behavior
TDF: theoretical domains framework
