# Supplementary material for: Psychosocial Factors of Health Professionals’ Intention to Use a Decision Aid for Down Syndrome Screening: Cross-Sectional Quantitative Study
Source: J Med Internet Res. 2018 Apr 25;20(4):e114. doi: 10.2196/jmir.9036 (PMC5943629; doi:10.2196/jmir.9036)
Supplement: Multimedia Appendix 2 [file jmir_v20i4e114_app2.pdf]

Multimedia Appendix 2

Psychosocial Factors of Health Professionals' Intention to Use a Decision Aid for Down Syndrome Screening: Cross-Sectional Quantitative Study, A.Rahimi et al.

Appendix 2

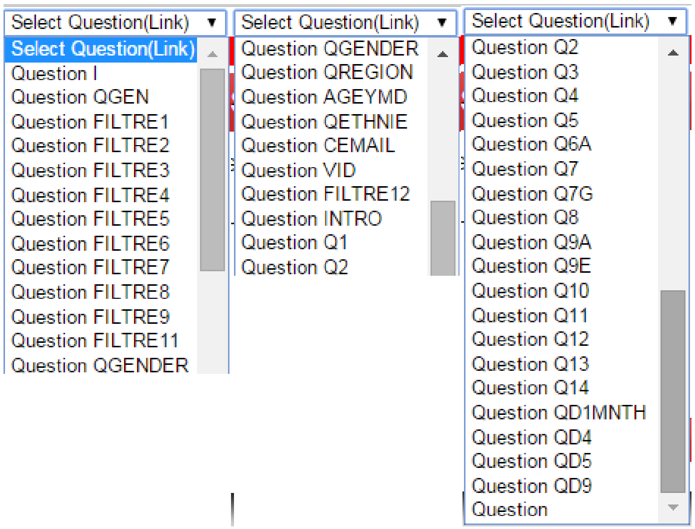

Select Question(Link)
Select Question(Link)
Question I
Question QGEN
Question FILTRE1
Question FILTRE2
Question FILTRE3
Question FILTRE4
Question FILTRE5
Question FILTRE6
Question FILTRE7
Question FILTRE8
Question FILTRE9
Question FILTRE11
Question QGENDER

Select Question(Link)
Question QGENDER
Question QREGION
Question AGEYMD
Question QETHNIE
Question CEMAIL
Question VID
Question FILTRE12
Question INTRO
Question Q1
Question Q2

Select Question(Link)
Question Q2
Question Q3
Question Q4
Question Q5
Question Q6A
Question Q7
Question Q7G
Question Q8
Question Q9A
Question Q9E
Question Q10
Question Q11
Question Q12
Question Q13
Question Q14
Question QD1MNTH
Question QD4
Question QD5
Question QD9
Question

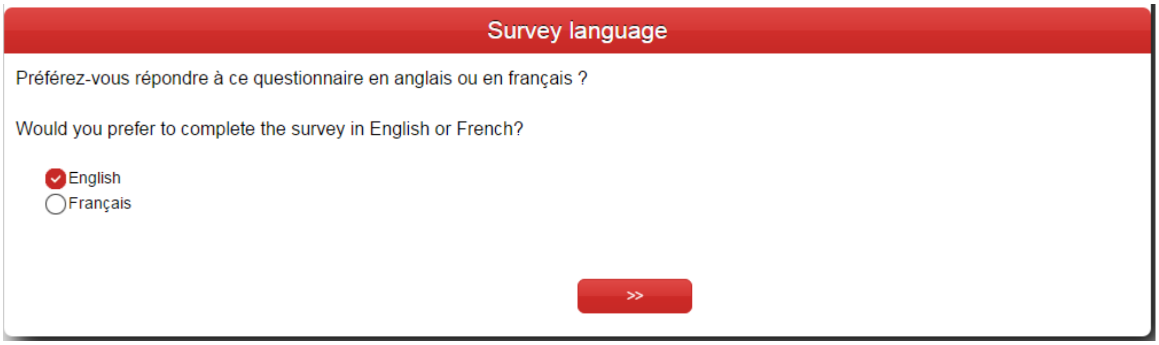

Survey language

Préférez-vous répondre à ce questionnaire en anglais ou en français ?

Would you prefer to complete the survey in English or French?

- English
- Français

>>

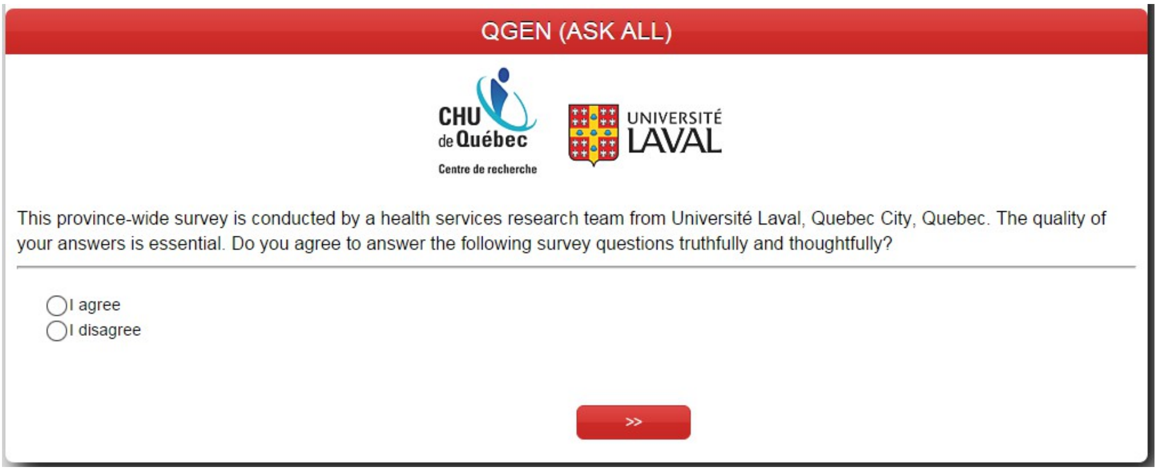

QGEN (ASK ALL)

CHU de Québec Centre de recherche | UNIVERSITÉ LAVAL

This province-wide survey is conducted by a health services research team from Université Laval, Quebec City, Quebec. The quality of your answers is essential. Do you agree to answer the following survey questions truthfully and thoughtfully?

- I agree
- I disagree

>>

Multimedia Appendix 2

Psychosocial Factors of Health Professionals' Intention to Use a Decision Aid for Down Syndrome Screening: Cross-Sectional Quantitative Study, A.Rahimi et al.

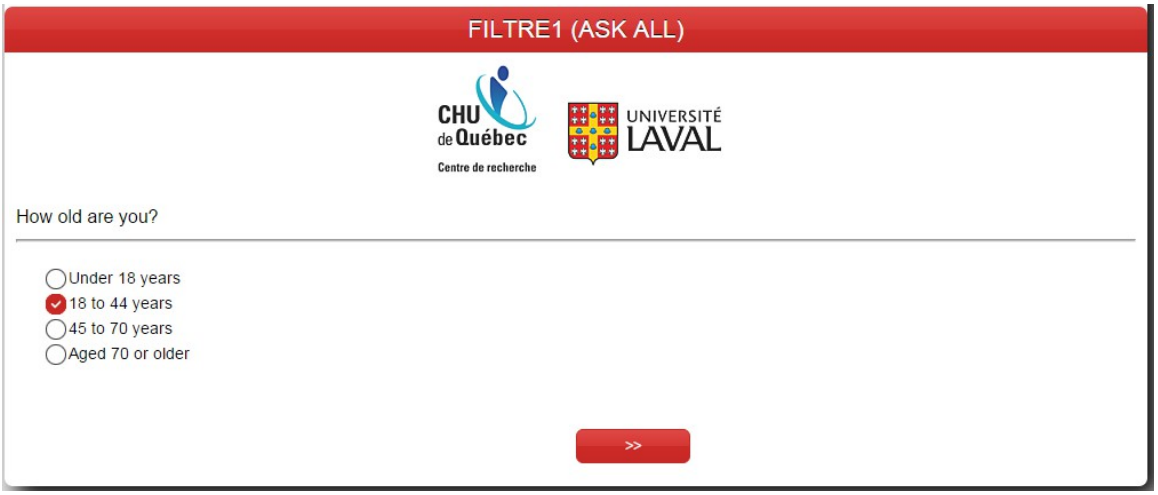

FILTRE1 (ASK ALL)

How old are you?

- Under 18 years
- 18 to 44 years
- 45 to 70 years
- Aged 70 or older

>>

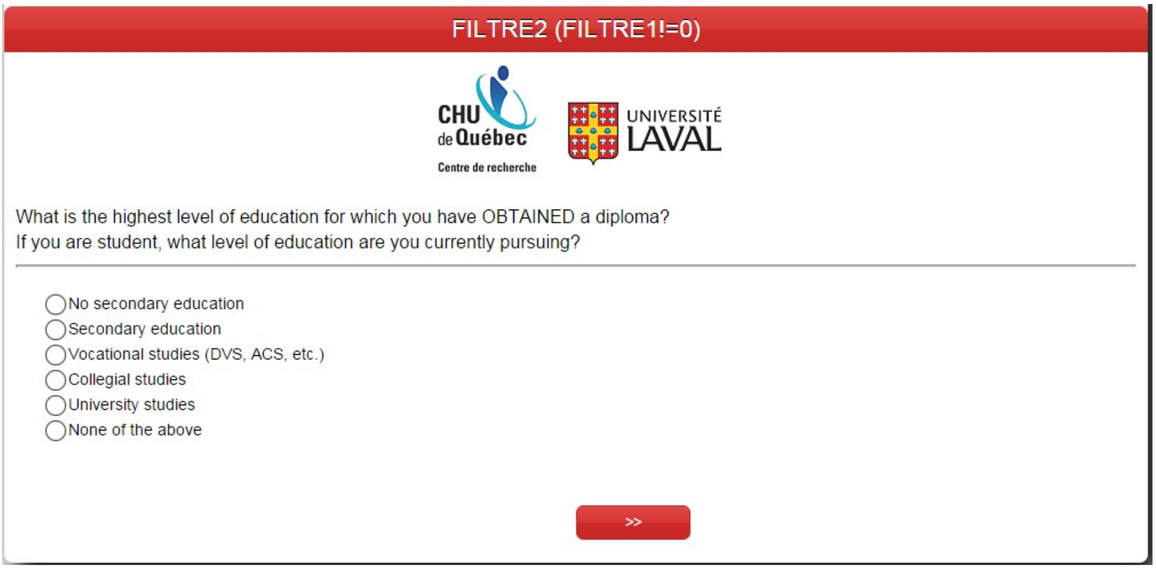

FILTRE2 (FILTRE1!=0)

What is the highest level of education for which you have OBTAINED a diploma?
If you are student, what level of education are you currently pursuing?

- No secondary education
- Secondary education
- Vocational studies (DVS, ACS, etc.)
- Collegial studies
- University studies
- None of the above

>>

Multimedia Appendix 2

Psychosocial Factors of Health Professionals' Intention to Use a Decision Aid for Down Syndrome Screening: Cross-Sectional Quantitative Study, A.Rahimi et al.

FILTRE3 (FILTRE2=5)

CHU de Québec Centre de recherche — UNIVERSITÉ LAVAL

What is your employment status?

- ◯ I am currently on a leave of absence because I am on preventive withdrawal; on maternity, paternity or parental leave; on sick leave; on sabbatical; other reason for absence, please specify: ________
- ◯ I have full-time employment, as a wage earner and/or a self-employed worker
- ◯ I have part-time employment, as a wage earner and/or a self-employed worker
- ◯ I am a student, with or without employment
- ◯ I am retired
- ◯ I am unemployed and I am not a student
- ◯ None of the above

>>

FILTRE4 (FILTRE3=2,3,4)

CHU de Québec Centre de recherche — UNIVERSITÉ LAVAL

What field of study did you choose in order to obtain your current employment?
If you are student, in which field are you studying?

- ◯ Administration and management
- ◯ Education
- ◯ Engineering
- ◯ Health sciences
- ◯ Human sciences
- ◯ Political science and law
- ◯ None of the above

>>

Multimedia Appendix 2

Psychosocial Factors of Health Professionals' Intention to Use a Decision Aid for Down Syndrome Screening: Cross-Sectional Quantitative Study, A.Rahimi et al.

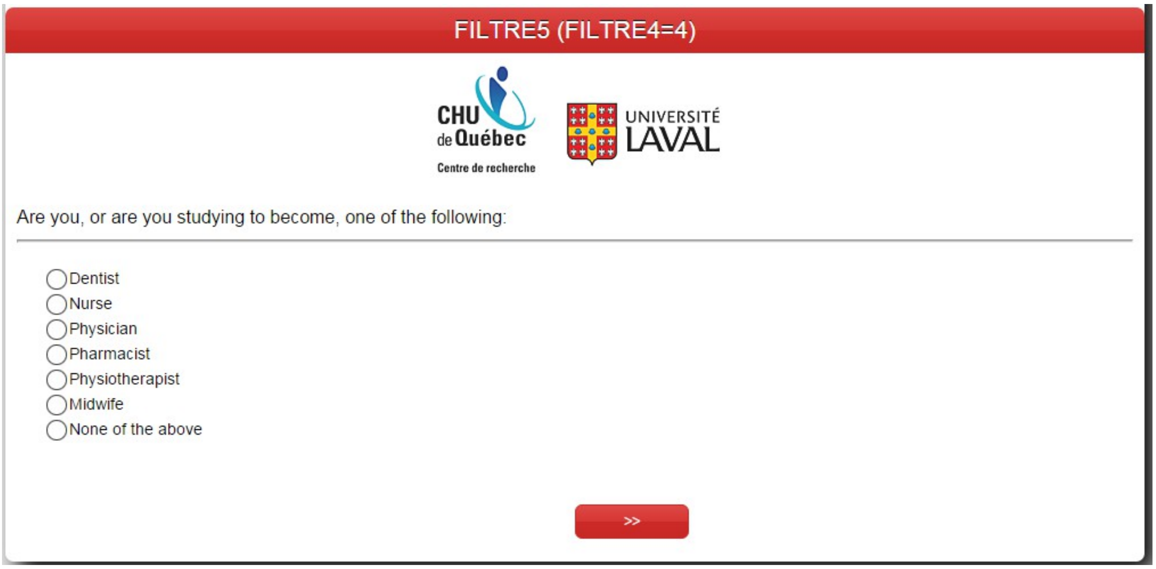

FILTRE5 (FILTRE4=4)

CHU de Québec Centre de recherche

UNIVERSITÉ LAVAL

Are you, or are you studying to become, one of the following:

- ◯ Dentist
- ◯ Nurse
- ◯ Physician
- ◯ Pharmacist
- ◯ Physiotherapist
- ◯ Midwife
- ◯ None of the above

>>

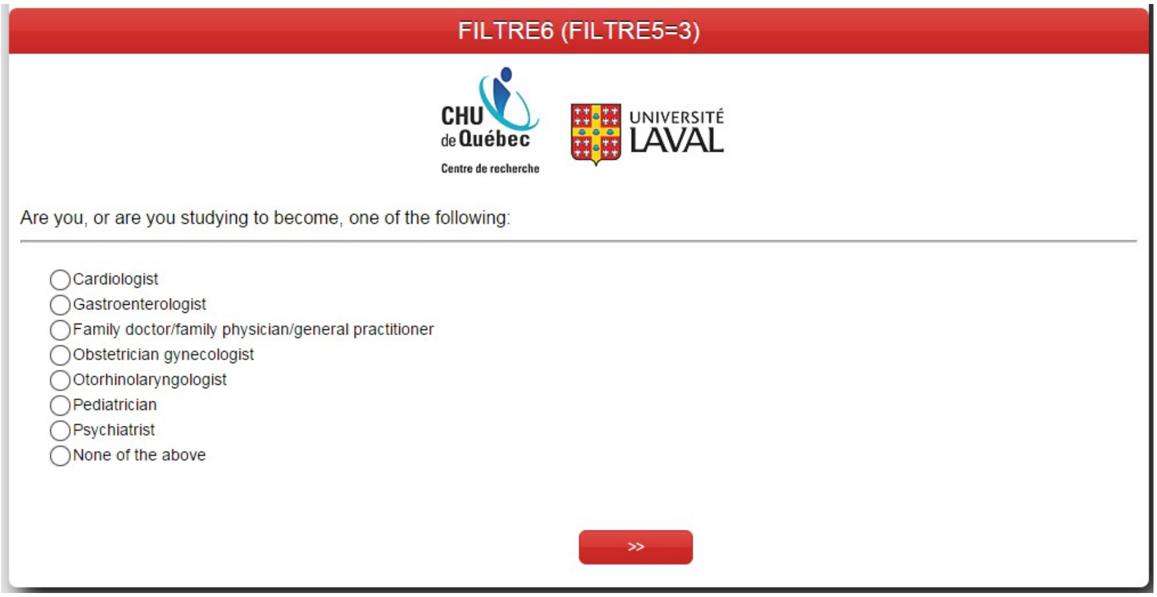

FILTRE6 (FILTRE5=3)

CHU de Québec Centre de recherche

UNIVERSITÉ LAVAL

Are you, or are you studying to become, one of the following:

- ◯ Cardiologist
- ◯ Gastroenterologist
- ◯ Family doctor/family physician/general practitioner
- ◯ Obstetrician gynecologist
- ◯ Otorhinolaryngologist
- ◯ Pediatrician
- ◯ Psychiatrist
- ◯ None of the above

>>

FILTRE7 (FILTRE6=3,4)

CHU de Québec Centre de recherche — UNIVERSITÉ LAVAL

Please check whichever applies to you:

- ◯ I am doing my pre-externship (classroom) in medicine
- ◯ I am doing my externship (clinical training) in medicine
- ◯ I am doing my internship (specialization) in medicine
- ◯ I am a physician (I have obtained my license to practice)

>>

FILTRE8 (FILTRE5=6)

CHU de Québec Centre de recherche — UNIVERSITÉ LAVAL

Please check whichever applies to you:

- ◯ I haven't started yet my internship (last clinical training)
- ◯ I am doing my internship (last clinical training)
- ◯ I am a midwife (I have obtained my license to practice)

>>

FILTRE9 (FILTRE7=3,4 OR FILTRE8=2,3)

CHU de Québec Centre de recherche — UNIVERSITÉ LAVAL

What type(s) of patients/clients have consulted you in the last month?

Please check all that apply to you:

- ☐ Newborns
- ☐ Babies
- ☐ Children
- ☐ Teenagers
- ☐ Adults
- ☐ Pregnant women
- ☐ Seniors

>>

Multimedia Appendix 2

Psychosocial Factors of Health Professionals' Intention to Use a Decision Aid for Down Syndrome Screening: Cross-Sectional Quantitative Study, A.Rahimi et al.

FILTRE11 (FILTRE10=2-6)

CHU de Québec Centre de recherche — UNIVERSITÉ LAVAL

In the past two years, have you participated in a research project asking you to view this video (see the three representative images below)?

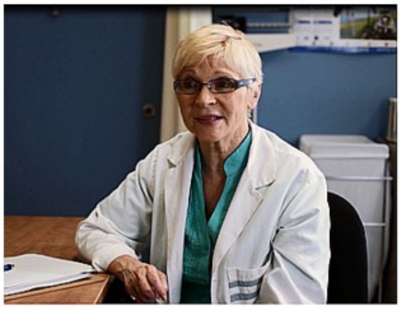
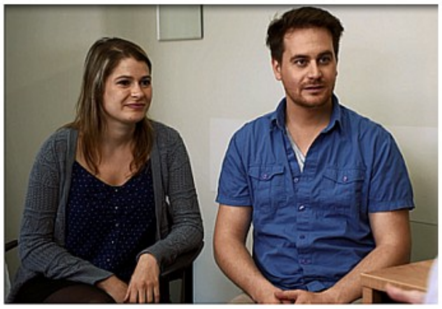

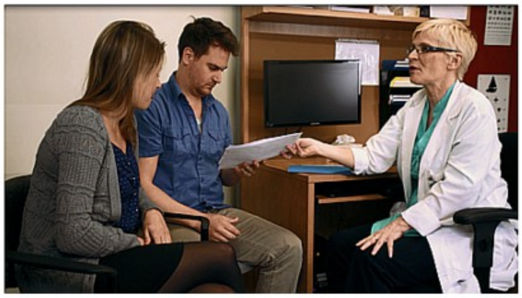

- ◯ I have seen this video
- ◯ I have never seen this video
- ◯ I am not sure if I have already seen this video

QGENDER (FILTRE11=2,3)

CHU de Québec Centre de recherche — UNIVERSITÉ LAVAL

Are you...

- ◯ A man
- ◯ A woman

>>

Multimedia Appendix 2

Psychosocial Factors of Health Professionals' Intention to Use a Decision Aid for Down Syndrome Screening: Cross-Sectional Quantitative Study, A.Rahimi et al.

QREGION (ASK ALL)

Which region of Quebec do you live in?

- ◯ Bas-Saint-Laurent
- ◯ Saguenay-Lac-Saint-Jean
- ◯ Capitale-Nationale
- ◯ Mauricie
- ◯ Estrie
- ◯ Montréal
- ◯ Outaouais
- ◯ Abitibi-Témiscamingue
- ◯ Côte-Nord
- ◯ Nord-du-Québec
- ◯ Gaspésie/Îles-de-la-Madeleine
- ◯ Chaudière-Appalaches
- ◯ Laval
- ◯ Lanaudière
- ◯ Laurentides
- ◯ Montérégie
- ◯ Centre-du-Québec
- ◯ Other, please specify: ______
- ◯ I don't know

>>

AGEYMD (Ask all)

What is your date of birth?

Please indicate your Year of birth :

Please select your answer ▼

>>

Multimedia Appendix 2

Psychosocial Factors of Health Professionals' Intention to Use a Decision Aid for Down Syndrome Screening: Cross-Sectional Quantitative Study, A.Rahimi et al.

QETHNIE (ASK ALL)

CHU de Québec Centre de recherche — Université Laval

What is your ethnic background?

Please check all that apply to you

- ☐ White/Caucasian
- ☐ Black/African/Afro-American, etc.
- ☐ Native/First Nations
- ☐ Latin American (Mexico, Chile, Costa Rica, etc.)
- ☐ Arab (Middle East, Maghreb, etc.)
- ☐ South Asian (India, Bangladesh, Pakistan, Sri Lanka, etc.)
- ☐ Southeast Asian (Vietnam, Cambodia, Malaysia, Laos, etc.)
- ☐ West Asian (Iran, Afghanistan, etc.)
- ☐ Chinese
- ☐ Filipino
- ☐ Korean
- ☐ Japanese
- ☐ Other, please specify: ______
- ◯ I would rather not answer

>>

CEMAIL - ASK ALL

CHU de Québec Centre de recherche — Université Laval

Good news! You are eligible to participate in this study. Thank you very much.

You will first be invited to watch a 10-minute video. You must watch the whole video and then complete the questionnaire (about 15 minutes).

To get the $50 compensation, you must complete the full questionnaire. Subsequently we will ask you to provide some personal information, which will remain confidential, in order to mail your $50 cheque. You can also refuse the $50 compensation by checking the appropriate box at the end of the survey, so you will not have to give these information.

You must complete the full questionnaire as soon as possible because the link to this survey will be deactivated when the number of desired respondents is reached. If you want to complete the survey in more than one session, please provide a valid email address and we will send you a personal internet link to the survey.

please enter your email address:

Valid email address: ______

☐ I want to complete the survey in a single session.

Please click on the following arrow to continue.

>>

Multimedia Appendix 2

Psychosocial Factors of Health Professionals' Intention to Use a Decision Aid for Down Syndrome Screening: Cross-Sectional Quantitative Study, A.Rahimi et al.

VID - ASK ALL

Health professionals who monitor pregnancies must inform pregnant women of available screening tests for Down syndrome (or trisomy 21). The use of a **Decision Aid** could increase the active involvement of pregnant women in making an informed and shared decision, which respects their values and preferences, regarding the choice of whether or not to have prenatal screening for Down syndrome.

Please watch this video that shows the optimal use of a **Decision Aid (four- page written document)** that intends to help the couple decide whether or not to have prenatal screening for Down syndrome while getting the support and advice of their family physician.

The goal of the following questionnaire is to **find out your opinion on the use of this Decision Aid if you had to use it in the future**.

Please click on " Play : ► " to start the video.

11:02

After watching the whole video, an arrow will appear below and you will be able to start the questionnaire.
Please note that it is normal that you cannot select an option at the end of the video (deactivated function).

☐ I cannot see this video (technical difficulties)

FILTRE12 (FILTRE11=3)

CHU de Québec Centre de recherche — UNIVERSITÉ LAVAL

Because you were unsure, can you confirm whether or not you have already seen this video?

◯ I have seen this video before
◯ I had not seen this video until today

>>

Multimedia Appendix 2

Psychosocial Factors of Health Professionals' Intention to Use a Decision Aid for Down Syndrome Screening: Cross-Sectional Quantitative Study, A.Rahimi et al.

INTRO (ASK ALL)

CHU de Québec Centre de recherche — UNIVERSITÉ LAVAL

The video that you just watched showed the optimal use of a **Decision Aid (four-page written document)** that intends to help the couple decide whether or not to have prenatal screening for Down syndrome while getting the support and advice of their family physician.

The goal of the following questionnaire is **to find out your opinion on the use of this Decision Aid if you had to use it in the future**.

We are carrying out a similar project with pregnant women to find out their opinion. The objective is to introduce the routine use of a decision aid that is appropriate and helpful for all concerned.

Please note:

1. For each question, please **check the box** which best corresponds to your answer. There are no right or wrong answers. It is **your opinion** that interests us.
2. Certain statements may seem repetitive. This is due to the study's methodology. Please answer **all** questions.
3. It is important that you complete the whole questionnaire **yourself**, without any help.
4. By completing this survey, you give your **consent** to participate in this research project.
5. At the end of the questionnaire, we will ask you to answer a few socio-demographic questions.

Your participation is much appreciated, thank you.

Team of the Canada Research Chair in Implementation of Shared Decision Making in Primary Care. Dr. France Légaré, MD, PhD, clinician researcher and full professor, CHUQ Research Centre, Department of Family Medicine and Emergency Medicine, Faculty of Medicine, Université Laval, Quebec City, Quebec, Canada.

This project is funded by a research grant from Genome Canada and Genome Quebec and was approved by the ethical review boards of CHU de Québec, CSSS Vieille-Capitale and CSSS Alphonse-Desjardins.

To keep your responses confidential, the firm Leger will assign you a numerical code corresponding to your personal information to which they alone will have the key.

Please click on the following arrow to continue.

>>

Multimedia Appendix 2

Psychosocial Factors of Health Professionals' Intention to Use a Decision Aid for Down Syndrome Screening: Cross-Sectional Quantitative Study, A.Rahimi et al.

Q1 (ASK ALL)

CHU de Québec Centre de recherche — UNIVERSITÉ LAVAL

In the context of prenatal screening for Down syndrome, using a decision aid...

	Very unlikely	Somewhat unlikely	Slightly unlikely	Neither unlikely, nor likely	Slightly likely	Somewhat likely	Very likely
...would help pregnant women reflect upon the issue.	○	○	○	○	○	○	○
...would enable the pregnant woman to express her preferences.	○	○	○	○	○	○	○
...would promote decision making.	○	○	○	○	○	○	○
...might require more time than planned for the consultation.	○	○	○	○	○	○	○
...would be a source of relevant information.	○	○	○	○	○	○	○
...could make the pregnant woman anxious.	○	○	○	○	○	○	○

>>

Q2 (ASK ALL)

CHU de Québec Centre de recherche — UNIVERSITÉ LAVAL

In the context of prenatal screening for Down syndrome, it would be easier for me to use a decision aid...

	Strongly disagree	Somewhat disagree	Slightly disagree	Neither disagree, nor agree	Slightly agree	Somewhat agree	Strongly agree
...if it was available in my consulting room.	○	○	○	○	○	○	○
...if it was presented to the pregnant woman by a nurse before the consultation.	○	○	○	○	○	○	○
...if it was given to the pregnant woman before the consultation.	○	○	○	○	○	○	○
...if I had prior training on how to use it.	○	○	○	○	○	○	○

>>

Multimedia Appendix 2

Psychosocial Factors of Health Professionals' Intention to Use a Decision Aid for Down Syndrome Screening: Cross-Sectional Quantitative Study, A.Rahimi et al.

Q3 (ASK ALL)

CHU de Québec Centre de recherche

UNIVERSITÉ LAVAL

In the context of prenatal screening for Down syndrome, the following people would approve/disapprove of my using a decision aid:

	Would strongly disapprove	Would moderately disapprove	Would slightly disapprove	Would neither disapprove, nor approve	Would slightly approve	Would moderately approve	Would strongly approve
my colleagues	○	○	○	○	○	○	○
my family	○	○	○	○	○	○	○
my patients/clients	○	○	○	○	○	○	○
public organization(s)	○	○	○	○	○	○	○
a colleague with extensive experience	○	○	○	○	○	○	○

>>

Q4 (ASK ALL)

CHU de Québec Centre de recherche

UNIVERSITÉ LAVAL

In the context of prenatal screening for Down syndrome, in your view, what percentage of your colleagues would use a decision aid?

None 0%	A quarter 25%	Half 50%	Three quarters 75%	All 100%
○	○	○	○	○

>>

Multimedia Appendix 2

Psychosocial Factors of Health Professionals' Intention to Use a Decision Aid for Down Syndrome Screening: Cross-Sectional Quantitative Study, A.Rahimi et al.

Q5 (ASK ALL)

CHU de Québec Centre de recherche — Université Laval

In the context of prenatal screening for Down syndrome, ...

	Very unlikely	Somewhat unlikely	Slightly unlikely	Neither unlikely, nor likely	Slightly likely	Somewhat likely	Very likely
...I would regret NOT having used a decision aid.	○	○	○	○	○	○	○
...my patients/clients' uncertainty would encourage me to use a decision aid.	○	○	○	○	○	○	○
...I believe that most of my colleagues would use a decision aid.	○	○	○	○	○	○	○
...the need to make a decision with my patient/client would motivate me to use a decision aid.	○	○	○	○	○	○	○
...I would be fine with NOT using a decision aid.	○	○	○	○	○	○	○
...if I wanted to, I could easily use a decision aid.	○	○	○	○	○	○	○

>>

Multimedia Appendix 2

Psychosocial Factors of Health Professionals' Intention to Use a Decision Aid for Down Syndrome Screening: Cross-Sectional Quantitative Study, A.Rahimi et al.

CHU de Québec Centre de recherche

UNIVERSITÉ LAVAL

In the context of prenatal screening for Down syndrome, using a decision aid would be...

Very difficult	Somewhat difficult	Slightly difficult	Neither difficult, nor easy	Slightly easy	Somewhat easy	Very easy
○	○	○	○	○	○	○

Very detrimental	Somewhat detrimental	Slightly detrimental	Neither detrimental, nor beneficial	Slightly beneficial	Somewhat beneficial	Very beneficial
○	○	○	○	○	○	○

Very depreciating	Somewhat depreciating	Slightly depreciating	Neither depreciating, nor gratifying	Slightly gratifying	Somewhat gratifying	Very gratifying
○	○	○	○	○	○	○

Very optional	Somewhat optional	Slightly optional	Neither optional, nor essential	Slightly essential	Somewhat essential	Very essential
○	○	○	○	○	○	○

Very useless	Somewhat useless	Slightly useless	Neither useless, nor useful	Slightly useful	Somewhat useful	Very useful
○	○	○	○	○	○	○

Very unpleasant	Somewhat unpleasant	Slightly unpleasant	Neither unpleasant, nor pleasant	Slightly pleasant	Somewhat pleasant	Very pleasant
○	○	○	○	○	○	○

>>

Q7 (ASK ALL)

CHU de Québec Centre de recherche · UNIVERSITÉ LAVAL

In the context of prenatal screening for Down syndrome, ...

	Strongly disagree	Somewhat disagree	Slightly disagree	Neither disagree, nor agree	Slightly agree	Somewhat agree	Strongly agree
... using a decision aid would depend only on me	○	○	○	○	○	○	○
...most people who are important to me would recommend that I use a decision aid.	○	○	○	○	○	○	○
... I would feel that I am able to use a decision aid.	○	○	○	○	○	○	○
...the people who are most important to me would think that I should use a decision aid.	○	○	○	○	○	○	○
... I believe that many of my colleagues would use a decision aid.	○	○	○	○	○	○	○
... it would be up to me to use a decision aid.	○	○	○	○	○	○	○

>>

CHU de Québec Centre de recherche · UNIVERSITÉ LAVAL

In the context of prenatal screening for Down syndrome, ...:

... If I used a decision aid, most of the people who are important to me...

Would strongly disapprove	Would moderately disapprove	Would slightly disapprove	Would neither disapprove, nor approve	Would slightly approve	Would moderately approve	Would strongly approve
○	○	○	○	○	○	○

... to what extent do you feel that you have control over the decision to use a decision aid?

Very uncontrollable	Somewhat uncontrollable	Slightly uncontrollable	Neither uncontrollable, nor controllable	Slightly controllable	Somewhat controllable	Very controllable
○	○	○	○	○	○	○

>>

Multimedia Appendix 2

Psychosocial Factors of Health Professionals' Intention to Use a Decision Aid for Down Syndrome Screening: Cross-Sectional Quantitative Study, A.Rahimi et al.

Q8 (ASK ALL)

CHU de Québec Centre de recherche — Université Laval

In the context of prenatal screening for Down syndrome, as a health professional, ...

	Strongly disagree	Somewhat disagree	Slightly disagree	Neither disagree, nor agree	Slightly agree	Somewhat agree	Strongly agree
... I consider that it would be appropriate for me to use a decision aid.	○	○	○	○	○	○	○
... I consider it to be my role to use a decision aid.	○	○	○	○	○	○	○
... I consider that it would be necessary for me to use a decision aid.	○	○	○	○	○	○	○
... I consider that it is my duty to present the decision aid to my patients/clients.	○	○	○	○	○	○	○

>>

CHU de Québec Centre de recherche

UNIVERSITÉ LAVAL

In the context of prenatal screening for Down syndrome ...

... I plan to use a decision aid.

Very unlikely	Somewhat unlikely	Slightly unlikely	Neither unlikely, nor likely	Slightly likely	Somewhat likely	Very likely
○	○	○	○	○	○	○

... using a decision aid would be in keeping with my moral values.

Strongly disagree	Somewhat disagree	Slightly disagree	Neither disagree, nor agree	Slightly agree	Somewhat agree	Strongly agree
○	○	○	○	○	○	○

... even if it was difficult, I would be able to use a decision aid.

Very unlikely	Somewhat unlikely	Slightly unlikely	Neither unlikely, nor likely	Slightly likely	Somewhat likely	Very likely
○	○	○	○	○	○	○

... using a decision aid would be in keeping with my principles.

Strongly disagree	Somewhat disagree	Slightly disagree	Neither disagree, nor agree	Slightly agree	Somewhat agree	Strongly agree
○	○	○	○	○	○	○

>>

Multimedia Appendix 2

Psychosocial Factors of Health Professionals' Intention to Use a Decision Aid for Down Syndrome Screening: Cross-Sectional Quantitative Study, A.Rahimi et al.

Q9E (ASK ALL)

CHU de Québec Centre de recherche

UNIVERSITÉ LAVAL

In the context of prenatal screening for Down syndrome ...

	Very unlikely	Somewhat unlikely	Slightly unlikely	Neither unlikely, nor likely	Slightly likely	Somewhat likely	Very likely
... I intend to use a decision aid.	○	○	○	○	○	○	○

	Strongly disagree	Somewhat disagree	Slightly disagree	Neither disagree, nor agree	Slightly agree	Somewhat agree	Strongly agree
...my personal values would lead me to use a decision aid.	○	○	○	○	○	○	○
...using a decision aid would be ethically acceptable for me.	○	○	○	○	○	○	○
... I believe that most of my colleagues would consider that using a decision aid is a good practice	○	○	○	○	○	○	○

	Very low	Somewhat low	Slightly low	Neither low, nor strong	Slightly strong	Somewhat strong	Very strong
... the likelihood of my using a decision aid is:	○	○	○	○	○	○	○

>>

Q10 (ASK ALL)

CHU de Québec Centre de recherche

UNIVERSITÉ LAVAL

In the context of prenatal screening for Down syndrome, I would feel capable of using a decision aid...

	Strongly disagree	Somewhat disagree	Slightly disagree	Neither disagree, nor agree	Slightly agree	Somewhat agree	Strongly agree
...even if I do NOT have easy access to it.	○	○	○	○	○	○	○
...even if it is only available in paper format.	○	○	○	○	○	○	○
...even if this prolonged the consultation process.	○	○	○	○	○	○	○

>>

Multimedia Appendix 2

Psychosocial Factors of Health Professionals' Intention to Use a Decision Aid for Down Syndrome Screening: Cross-Sectional Quantitative Study, A.Rahimi et al.

Q11 (ASK ALL)

CHU de Québec Centre de recherche | UNIVERSITÉ LAVAL

In the context of prenatal screening for Down syndrome, ...

	Very unlikely	Somewhat unlikely	Slightly unlikely	Neither unlikely, nor likely	Slightly likely	Somewhat likely	Very likely
...using a decision aid would give me satisfaction.	○	○	○	○	○	○	○
... I would feel comfortable using a decision aid.	○	○	○	○	○	○	○
...using a decision aid would reassure me.	○	○	○	○	○	○	○
...using a decision aid would give me confidence.	○	○	○	○	○	○	○

>>

Q12 (ASK ALL)

CHU de Québec Centre de recherche | UNIVERSITÉ LAVAL

In the context of prenatal screening for Down syndrome, I would use a decision aid if it respected the following elements:

	Strongly disagree	Somewhat disagree	Slightly disagree	Neither disagree, nor agree	Slightly agree	Somewhat agree	Strongly agree
It must include information on prenatal tests offered in the private sector.	○	○	○	○	○	○	○
It must not be biased in favor of one decision over the other.	○	○	○	○	○	○	○
It must respect the cultural, religious and ethnic values of my patients/clients.	○	○	○	○	○	○	○
It must include information about the Non-Invasive Prenatal Test (NIPT) that analyzes the DNA of the foetus in the blood of the pregnant woman.	○	○	○	○	○	○	○
It must be properly used during a single pregnancy follow-up appointment.	○	○	○	○	○	○	○

>>

Multimedia Appendix 2

Psychosocial Factors of Health Professionals' Intention to Use a Decision Aid for Down Syndrome Screening: Cross-Sectional Quantitative Study, A.Rahimi et al.

Q13 (ASK ALL)

CHU de Québec Centre de recherche — UNIVERSITÉ LAVAL

In the context of prenatal screening for Down syndrome, ...

	Yes	No
... I already knew a decision aid.	○	○
... I know the government tool.	○	○

I know a decision aid regarding another issue:

Yes	No
○	○

>>

QD1 (Ask all)

CHU de Québec Centre de recherche — UNIVERSITÉ LAVAL

When did you obtain your license to practice OR if you are student, when will you obtain it?

Month	year
Please select your answer ▾	Please select your answer ▾

>>

Multimedia Appendix 2

Psychosocial Factors of Health Professionals' Intention to Use a Decision Aid for Down Syndrome Screening: Cross-Sectional Quantitative Study, A.Rahimi et al.

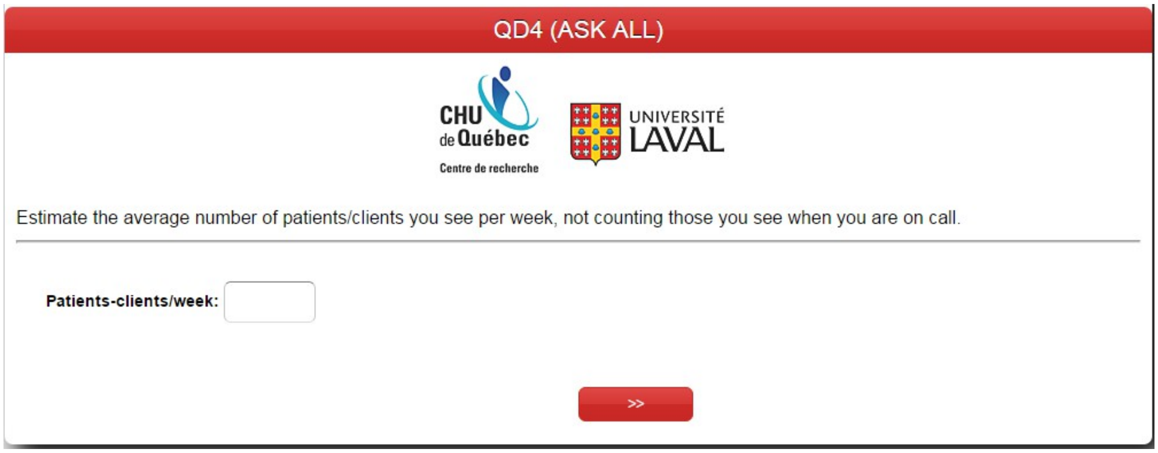

QD4 (ASK ALL)

CHU de Québec Centre de recherche · UNIVERSITÉ LAVAL

Estimate the average number of patients/clients you see per week, not counting those you see when you are on call.

Patients-clients/week:

>>

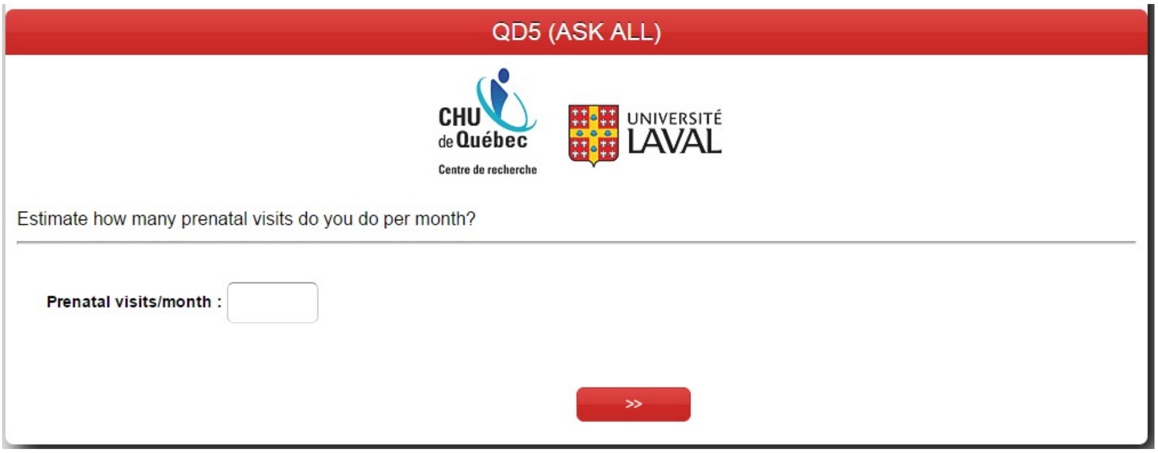

QD5 (ASK ALL)

CHU de Québec Centre de recherche · UNIVERSITÉ LAVAL

Estimate how many prenatal visits do you do per month?

Prenatal visits/month :

>>

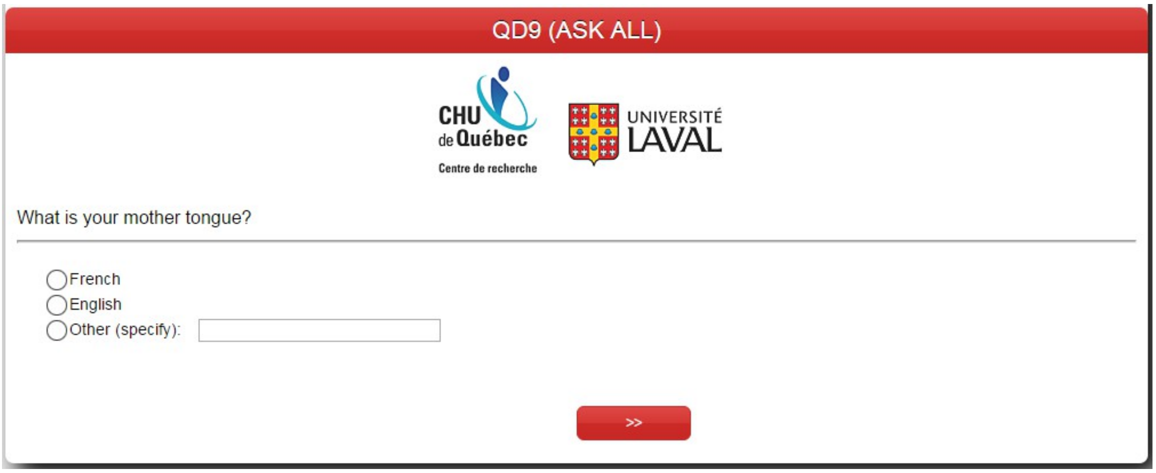

QD9 (ASK ALL)

CHU de Québec Centre de recherche · UNIVERSITÉ LAVAL

What is your mother tongue?

- French
- English
- Other (specify):

>>

Multimedia Appendix 2

Psychosocial Factors of Health Professionals' Intention to Use a Decision Aid for Down Syndrome Screening: Cross-Sectional Quantitative Study, A.Rahimi et al.

QADR0

CHU de Québec Centre de recherche — Université Laval

Thank you for your participation. Please provide your contact information in order to mail your $50 cheque. You will receive it in the coming weeks. Be assured that your personal information will be kept strictly confidential.

- ○ I refuse the $50 compensation.
- ✓ I want to get the $50 compensation

First name	
Last name	
Address Line 1	
Address Line 2	
City	
Province	
Postal Code:	
Permit number (please write 999 if ongoing)	

>>

MessComplete

CHU de Québec Centre de recherche — Université Laval

Your participation is much appreciated, thank you. If you have any questions or comments, please contact Johannie Lépine (PhD), post-doctoral fellow in public health.

Email:Johannie.Lepine.1@ulaval.ca

Phone: 418-525-4444 (Ext. 53701)

>>

Multimedia Appendix 2

Psychosocial Factors of Health Professionals' Intention to Use a Decision Aid for Down Syndrome Screening: Cross-Sectional Quantitative Study, A.Rahimi et al.

Canada research chair in implementation of shared decision making in primary care

| Contact us | Site map | KT Canada | Faculté de médecine | Disclaimer |

Français

UNIVERSITÉ LAVAL

Home page | The Chair | Research team | Research | PBRN | List of SDM programs | Decision aids | Tool box | Training | Useful links | Career |

Home page » Research » Projects » PEGASUS

Projects

- PEGASUS
- Continuing Prof. Dev.
- Cochrane systematic reviews
- Decision+
- Interprofessional approaches
- International collaboration
- Professional development
- Exackte 2
- Genetics
- UETMIS
- KT-Canada

PEGASUS: PErsonalized Genomics for prenatal Aneuploidy Screening USing maternal blood

Each year, 450,000 Canadian women become pregnant and, as a result of their participation in prenatal screening for Down syndrome, approximately 10,000 of them will have an amniocentesis (i.e. sampling of liquid surrounding the fetus) and of those, 315 will be found to carry a baby with Down syndrome and 70 normal pregnancies will be lost from complications of the procedure.

It has been discovered recently that, during pregnancy, there is fetal DNA in maternal blood in sufficient quantities to be analyzed and methods have been proposed to detect the presence of a fetus with Down syndrome using maternal blood. The introduction of genomic blood testing as proposed in the context of this project could lead to increased detection of Down syndrome, less invasive screening with 9,700 amniocenteses avoided each year in Canada, improving the peace of mind of pregnant women, and preventing the accidental loss of 70 normal fetuses, at a lower overall cost than current practice.

However, these methods still need to be validated before being appropriately introduced in routine care. This project proposes to carry out an independent study that will validate the performance and utility of these new genomic technologies for screening in pregnant women using maternal blood. The team of researchers will identify an evidence-based cost-effective approach for implementation of this new technology in the Canadian health care system. They will develop decision-making tools that will assist couples in making informed decisions, as well as educational tools for health care professionals, all integral components of the implementation of genomics-based non-invasive prenatal diagnosis.

The deliverables of this project will enable decision makers–pregnant women and their partner–to make informed choices pertaining to prenatal genetic screening and diagnosis, such as screening for Down syndrome, and to reduce the risk to pregnancies associated with amniocentesis.

Official website

Print | Send to a friend | Disclaimer

Last site update 2016-01-15
Last page update 2015-01-08

http://www.decision.chaire.fmed.ulaval.ca/en/research/projects/pegasus/
